# Extracellular matrix contribution to disease progression and dysfunction in myopathy

**DOI:** 10.1152/ajpcell.00182.2023

**Published:** 2023-09-25

**Authors:** Ashlee M. Long, GaHyun Lee, Alexis R. Demonbreun, Elizabeth M. McNally

**Affiliations:** ^1^Center for Genetic Medicine, Northwestern University Feinberg School of Medicine, Chicago, Illinois, United States; ^2^Department of Pharmacology, Northwestern University Feinberg School of Medicine, Chicago, Illinois, United States

**Keywords:** acellular myoscaffolds, decellularization, fibrosis, heart, muscle

## Abstract

Myopathic processes affect skeletal muscle and heart. In the muscular dystrophies, which are a subset of myopathies, muscle cells are gradually replaced by fibrosis and fat, impairing muscle function as well as regeneration and repair. In addition to skeletal muscle, these genetic disorders often also affect the heart, where fibrofatty infiltration progressively accumulates in the myocardium, impairing heart function. Although considerable effort has focused on gene-corrective and gene-replacement approaches to stabilize myofibers and cardiomyocytes, the continual and ongoing deposition of extracellular matrix itself contributes to tissue and organ dysfunction. Transcriptomic and proteomic profiling, along with high-resolution imaging and biophysical measurements, have been applied to define extracellular matrix components and their role in contributing to cardiac and skeletal muscle weakness. More recently, decellularization methods have been adapted to an on-slide format to preserve the spatial geography of the extracellular matrix, allowing new insight into matrix remodeling and its direct role in suppressing regeneration in muscle. This review highlights recent literature with focus on the extracellular matrix and molecular mechanisms that contribute to muscle and heart fibrotic disorders. We will also compare how the myopathic matrix differs from healthy matrix, emphasizing how the pathological matrix contributes to disease.

## INTRODUCTION

**Figure 1.**
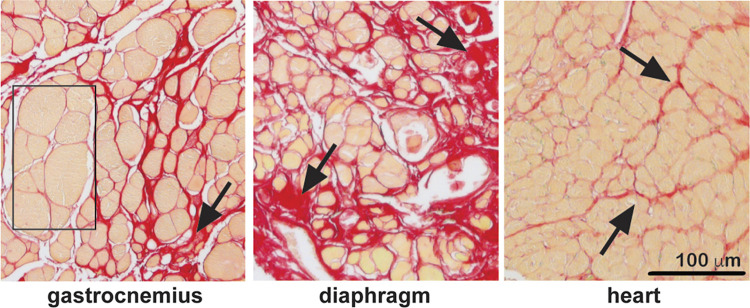
Sirius Red (collagen stain) demonstrates areas of fibrosis in gastrocnemius, diaphragm, and heart sections from dystrophin-deficient *mdx* mice, which serve as a model of Duchenne Muscular Dystrophy. The boxed area highlights a region of relatively intact muscle adjacent to regions of intense fibrosis (arrows). The diaphragm muscle is the most fibrotic muscle in this mouse model.

Myopathies are progressive diseases of striated muscle, which includes heart and skeletal muscle. Muscular dystrophies are a subset of the myopathies and are further defined by the replacement of skeletal muscle by fibrosis, inflammatory, and adipogenic cellular infiltrates. The muscular dystrophies are genetically heterogeneous arising from mutations in genes encoding extracellular, membrane-associated, and intracellular proteins (1). The dystrophin complex is a target for genetic muscular dystrophies since mutations affecting the integrity of this complex produce a fragile sarcolemma (2). Dystrophin is a wholly intracellular protein that adheres to transmembrane component β-dystroglycan. α-Dystroglycan, which is produced from the same gene as the β-dystroglycan subunit, is extracellular, heavily glycosylated, and binds directly to laminin-α2 in the extracellular matrix (ECM). Loss-of-function mutations in the gene encoding dystrophin result in Duchenne Muscular Dystrophy (DMD) and mutations in the sarcoglycan genes cause subtypes of Limb Girdle Muscular Dystrophy (LGMD) (Fig. 1) (1, 3). In-frame, internal genetic deletions, which leave intact dystrophin’s amino and carboxy termini, retain capacity to link the cytoskeleton to the ECM and result in the milder form disease Becker Muscular Dystrophy (4). The amount of dystrophin protein retained determines the degree of sarcolemma instability and, along with it, the degree of skeletal muscle degeneration. Cardiomyopathy can occur in dystrophin- and sarcoglycan-related muscular dystrophy, and it is also associated with fibrotic and adipogenic deposition in the ECM (Fig. 1). When the dystrophin complex is disrupted, cardiomyopathy, like skeletal muscle, is characterized by dysregulated intracellular calcium, which contributes to heart dysfunction (5). Cardiac fibrosis can be detected by noninvasive cardiac magnetic resonance imaging, and fibrosis predicts the decline of heart function in DMD (6). As in other forms of cardiac fibrosis, cardiac muscle injury activates cardiac fibroblasts and triggers ECM remodeling (7–9).

Although dystrophin disruption leads to a fragile sarcolemma, not all muscular dystrophies arise from this mechanism. For example, mutations in *DYSF*, the gene that encodes dysferlin, produce LGMD and Myoshi Myopathy, because of defective membrane repair (10). Studies on dysferlin contributed to the identification of a membrane repair complex that includes annexins A1, A2, and A6; EPS-15 homology domain proteins; and MG53/TRIM72 (11, 12). Dysferlin deficiency, with its disrupted membrane repair, also leads to the replacement of skeletal muscle tissue by fat and fibrosis. Muscle MRI highlights the initial involvement of the distal posterior muscles of the lower limbs with other muscle groups, such as the posterior muscles of the forearms, affected later in the disease process (13). How these two distinct pathological properties, sarcolemmal instability and defective membrane repair, differentially alter the ECM is not well defined.

**Figure 2.**
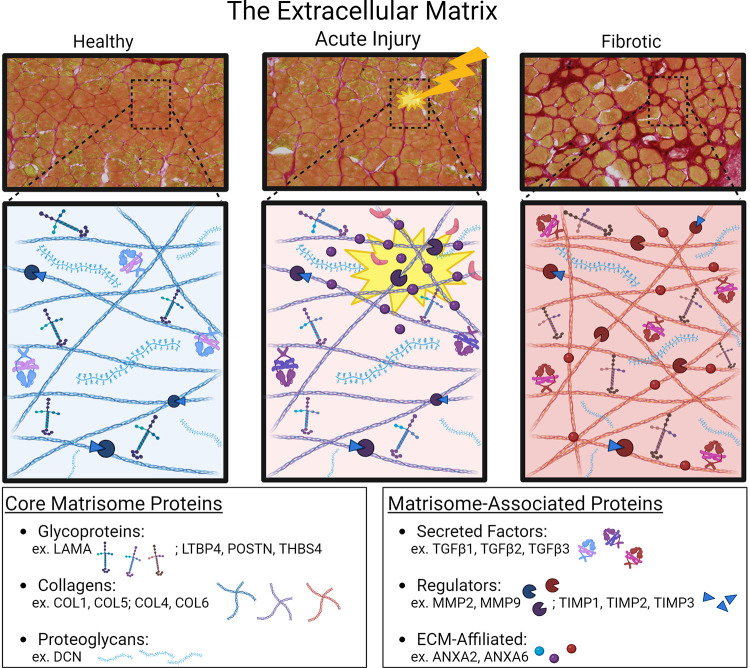
Schematic of the extracellular matrix and matrisome components important for muscle injury and repair. In skeletal muscle, acute injury leads to acute remodeling of the ECM, which can often fully resolve depending on the extent of the primary insult. In contrast, muscular dystrophy is characterized by ongoing degeneration concomitant with regeneration, which leads to progressive replacement of the myofibers by ECM. Figure created with BioRender.com. ECM, extra-cellular matrix.

In both skeletal and cardiac muscle, fibrosis is a pathological process that contributes to weakness, and in the case of skeletal muscle, impaired regeneration. Early transcriptomic profiling of genetically diverse dystrophic muscles emphasized the importance of ECM remodeling across nearly all muscular dystrophy subtypes (14). Bulk profiling approaches have been enhanced by single-cell transcriptomic profiling; single nuclear RNA sequencing of dystrophic muscle has contributed to identifying the range of cell types within the dystrophic milieu and demonstrated that many cells within dystrophic muscle produce ECM components (15). Proteomic profiling of dystrophic muscle provides additional support documenting ECM accumulation (16), which, like transcriptomic profiling, revealed shifts and excess collagen types, fibrinogen, and other components that regulate transforming growth factor (TGF)-β. A more recent study integrated transcriptomic and proteomic profiling and employed methods to focus more specifically on ECM proteins (17). In this study, the authors overexpressed the small tetraspanin protein sarcospan to reorganize the disrupted ECM in the *mdx* model of DMD. Sarcospan overexpression produced a remodeled transcriptome dominated by the gene ontology terms “extracellular matrix organization” and “extracellular structure organization.” Critically, dystrophic pathology is nonuniform across the disease muscle, with areas showing heavy fibrosis and other areas showing intact or even regenerated fibers (Fig. 2). The heterogeneous distribution of ECM in dystrophic muscle indicates the importance of integrating spatial information with protein content to fully dissect dystrophic pathology.

### The Extracellular Matrix Proteome in Normal and Diseased Muscle

In muscle, the ECM consists of three layers: the epimysium, the perimysium, and the endomysium. The epimysium sheath surrounds a muscle, whereas the perimysium encases a group of muscle fibers. The endomysium represents the interstitial layer between individual myofibers (18). These layers provide muscle structural support and contribute to force transmission. The normal ECM surrounding muscle and muscle fibers communicates with neighboring cells, stimulating cytoskeletal rearrangements and affecting cellular migration, intracellular signaling cascades, and gene expression (18). Proteomic catalogs of ECM composition have defined the “Matrisome,” which is separated into two distinct categories of “Core Matrisome Proteins” and “Matrisome-Associated Proteins.” Core Matrisome proteins include glycoproteins, collagens, and proteoglycans (19), whereas Matrisome-Associated proteins include secreted factors, ECM-affiliated proteins, and regulators. A study which specifically looked at the skeletal muscle matrisome identified over 2,100 proteins using a stepwise progression of protein extraction of phosphate-buffered saline, urea, and then guanidinium (20). This study identified 125 core matrisome proteins and 62 matrisome-associated proteins. A comparison of young and aged skeletal muscle identified that many ECM components increased with age, with the exception of annexin A6, which was decreased in aged muscle (20). Muscle ECM dynamically remodels in response to injury, which depending on the severity of injury and degree of regeneration, may fully resolve returning to baseline healthy ECM. In muscular dystrophy, there is asynchronous degeneration and regeneration, resulting in progressive dysfunctional ECM remodeling (Fig. 2).

### The Genesis of Fibrosis and Distinct Matrisome Profiles in Disease

Matrisome proteins accumulate in dystrophic and injured muscle. TGF-β, a secreted factor stored in the ECM, is a matrisome-associated protein and a main driver of fibrosis in many tissues and disease states including muscular dystrophy (14, 21). TGF-β is held in an inactive state in the matrix and requires activation to release the active peptide and engage TGF-β receptors. The mature TGF-β peptide remains associated with its propeptide forming a small latent complex. Latent TGF-β binding proteins (LTBPs) reside in the ECM where they bind TGF-β with its propeptide. The LTBPs are members of the fibrillin superfamily and contain multiple epidermal growth factor repeats and specialized 8-cysteine repeats. This large latent complex, defined as LTBP plus LAP-TGFβ, can also interact with other matrix elements like fibrillins, fibulins, or elastin, and LTBP matrix binding is mediated by its amino-terminus (22). LTBP4 was identified as a genetic modifier of muscular dystrophy in mice where a polymorphism in the LTBP4 hinge region correlated with muscle membrane stability and fibrosis (23). The polymorphism disrupts a hinge region in LTBP4, rendering it more susceptible to proteolysis, which is linked to release and activation of TGF-β. This polymorphism is found in the DBA/2J strain of mice, where it also intensifies the mouse model of DMD, the *mdx* mouse (24). Once activated, TGF-β engages cell surface receptors where it canonically mediates intracellular SMAD signaling to activate profibrotic gene expression leading to increased synthesis of collagens and other matrix proteins (25).

Fibroblasts isolated from DMD muscle biopsies demonstrate a profibrotic signature at baseline compared with fibroblasts from healthy controls (26). Furthermore, treatment with TGF-β1 further stimulated matrisome protein production of ECM regulators like matrix metalloproteinases (MMPs) and tissue inhibitors of metalloproteinases (TIMPs) (26). MMPs control matrix degradation, with TIMPs providing a counterbalance by inhibiting MMP activity. At baseline in DMD fibroblasts, MMP7 was increased while MMP1 and TIMP3 were decreased, and TGF-β1 treatment reversed these trends. Elevated TIMP1 protein levels are observed in dystrophic muscle and correlate with increased levels of TGF-β1 (27). Another study documented serum increases in MMP1, MMP7, MMP10, and lower TIMP4 in patients with DMD, and the authors correlated these findings with cardiac MRI measures finding that MMP7, in particular, correlated with cardiovascular disease severity, potentially indicating the use of these proteins as biomarkers (28). The balance of matrisome protein degradation and synthesis is dysregulated in DMD resulting in the progressive accumulation of ECM components and development of fibrosis.

Proteoglycan synthesis is increased in dystrophic skeletal muscles with accumulation of decorin (29). Periostin, another glycoprotein, is increased in dystrophic muscle and after an acute injury (30, 31). Deletion of *Postn* in a dystrophic mouse model partially rescued the dystrophic phenotype through altered TGF-β signaling resulting in reduced fibrosis (31). Thrombospondin-4, a core matrisome protein important for proper dystrophin-glycoprotein trafficking and integrin attachment complexes, is essential for muscle attachment, ECM assembly and sarcolemmal stabilization (32, 33). Loss of *Thbs4* led to more severe muscular dystrophy progression (33).

Annexin A6 is a membrane repair protein previously identified as a genetic modifier of muscular dystrophy (34). In mice, an *Anxa6* polymorphism encodes a truncated protein that exhibits a dominant-negative effect, disrupting membrane repair and correlating with more severe disease progression (34). With injury, annexin A6 forms a repair cap on the extracellular face of membrane disruption, and annexin A6 has been described as a matrix-associated protein. The role of annexin A6 in membrane repair is conserved in muscle, heart, and nerve cells (35). Recombinant annexin A6 can be delivered intracellularly or extracellularly where it acts in a protective manner in both acute muscle injury and in the setting of chronic muscle injury to promote muscle repair (36). Thus, matrix-associated proteins can contribute to muscle repair from an “outside-in” approach. There are number of matrix-associated proteins with membrane-associated roles like annexin A6, and the accumulation of some these proteins may directly influence muscle regeneration and muscle function through direct or indirect effects, for example, by also regulating the inflammatory response. Further studies are needed to better assess this aspect of ECM pathological remodeling.

### Assessing Cellular-ECM Interactions Using Decellularized Matrices

To date, much of the focus in studying the ECM has been on the cellular contribution and its role in secreting and depositing matrix components. However, ECM components also influence cellular function, which in dystrophic muscle extends to myoblasts, myofibers, fibro-adipogenic precursors, macrophages, and lymphocytes. Decellularization is the process of removing cellular components, and, by definition, decellularization removes cellular proteins, membranes, and nucleic acids but generally leaves many matrix and associated proteins intact. Decellularization has been widely applied in regenerative medicine since it yields a nonimmunogenic organ scaffold on which many different types of stem cells can be evaluated. There are multiple approaches to decellularization like application of surfactants (detergents), acids and bases, and enzymes and physical approaches like freeze/thaw or high hydrostatic pressure (37, 38). Most commonly, decellularization has been applied to whole organs using perfusion or immersion with the resultant decellularized ECM assessed for cell removal with preservation of matrisome components and retention of mechanical properties (37). The size of the organ may limit the uniform application of the decellularizing method. Sodium dodecyl sulfate (SDS) and Triton X-100 are often used chemical surfactants that lyse cells through phospholipid membrane disarrangement to remove cellular contents and leave behind the ECM (37, 38). Detergent-based decellularization may also expose protein epitopes normally masked by cellular content, which can, in some circumstances, enable better visualization or exposure of ECM components.

Methods have been optimized for quantitative ECM proteomics, and although conducted in tissues other than skeletal muscle, the results may be instructive for skeletal muscle fibrosis. Comparative quantitative proteomics between native lung and Triton/SDC (sodium deoxycholate)-decellularized ECM demonstrated near-native retention of core matrisome and matrisome-associated proteins in the decellularized lung ECM (39). Krasny et al. (40) performed a comparative proteomic study to assess the matrisome enrichment using different decellularization methodologies with SDS decellularization samples exhibiting matrisome-enriched samples with high purity. Similarly, after decellularizing human myocardial samples with SDS, Johnson et al. (41) identified over 200 distinct proteins, with cellular proteins representing less than 1% of total protein. Removal of the cells also enabled greater epitope accessibility and improved visualization and quantification of the matrisome. Several studies demonstrated increased collagen in acellular scaffolds after decellularization, and this increase in collagen likely reflects greater access of antibody-epitope binding (42–44). This method also lends itself to visualizing proteins while preserving their native distribution, allowing for spatial and colocalization interrogation of ECM proteins. Decellularization provides a useful method to illustrate not only matrisome composition but also its role in instructing cellular behavior.

Decellularized skeletal muscle can serve as a substrate for examining the behavior and differentiation potential of myoblasts or myoblast-like cells seeded onto these matrices. One study compared multiple methods for decellularization mouse skeletal muscle including SDS, Triton, or phospholipase A treatment, preferring phospholipase A (45). The authors used mouse and rat skeletal muscle decellularized scaffolds to evaluate C2C12 myoblast alignment and myotube formation, finding that muscle matrix plus fibronectin resulted in longer and more aligned myotubes, whereas myoblasts on collagen were less well organized. Another group evaluated chicken muscle decellularized scaffolds, confirming that these scaffolds afforded an improved capacity to study the interaction with C2C12 myoblasts (46). A decellularized ECM protocol has also been used to generate engineered heart tissues (EHT) created with decellularized laser-cut sheets of porcine myocardium (47). When reseeded with cardiomyocytes from neonatal rats or derived from stem cells, these EHTs retain anisotropic features and the matrix composition of the native myocardium, making them appropriate for mechanotransduction experiments (47). EHTs generated with normal, nondiseased cardiomyocytes seeded on the ECM of cardiomyopathic myocardium acquired abnormal contractile behavior, associated with poor relaxation, increased stiffness, and increased force development (48).

Collagen architecture directly regulates myoblast and muscle stem cell behavior. Collagen hydrogels varying in crosslinks, size, and alignment were seeded with C2C12 myoblasts or muscle stem cells (49). C2C12 myoblasts favored smaller collagen fibrils for differentiation, and neither muscle stem cells nor C2C12 myoblasts responded differentially to collagen alignment (49). Muscle stem cells had greater differentiation with fewer collagen crosslinks, similar to what was observed in the acellular myoscaffold study (44, 49). Using collagen-coated polyacrylamide to mimic different levels of stiffness, Loomis et al. (50) examined the behavior of fibro-adipogenic progenitors from muscle. These cells, which are drivers of muscle fibrosis, demonstrated increased activation to myofibroblasts on stiffer matrices, which was dependent on YAP, a downstream effector of the Hippo signaling pathway (50). In addition, on stiff substrates, YAP has also been previously demonstrated to participate in a feedback loop, resulting in enhanced MMP7 expression (51). This same pathway has been implicated in the aging muscle matrix (52)

### Evaluating ECM-Cellular Cross Talk in Myopathies

**Figure 3.**
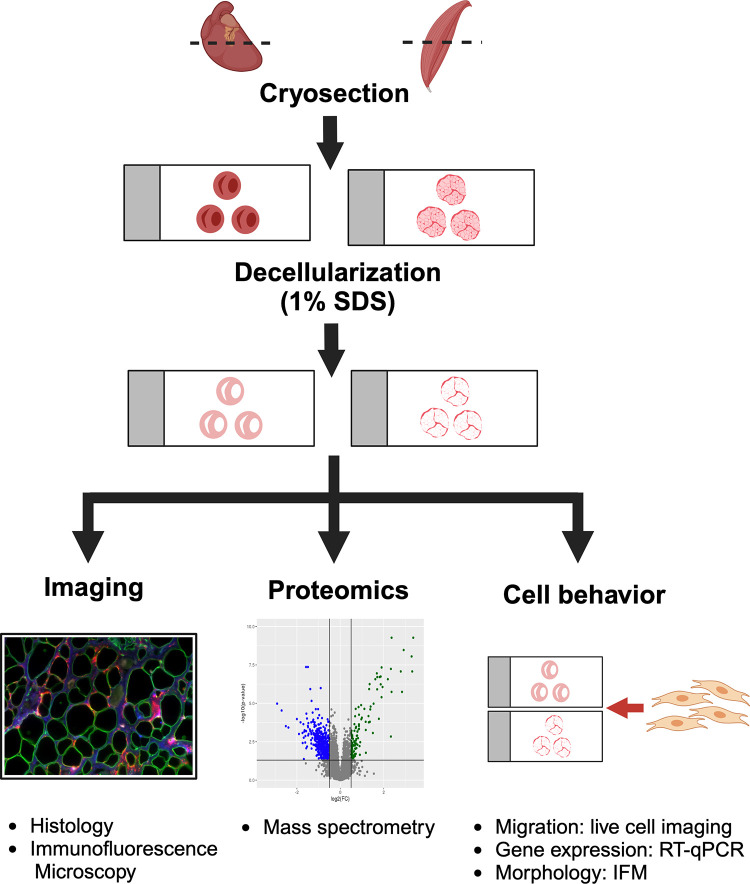
Diagram of the on-slide decellularization process for studying the ECM and its potential downstream applications. Figure created with BioRender.com. ECM, extracellular matrix.

Recently, Stearns-Reider et al. (44) optimized the on-slide decellularization method for the study of dystrophic muscle and its role on stem cell function. The inhomogeneity of fibrosis across dystrophic muscle lends itself well to on-slide decellularization since this method preserves the architecture allowing the authors to compare cellular behavior on scarred versus less-scarred areas of dystrophic muscle. Dystrophic mouse muscles were cryosectioned, mounted, and decellularized with 1% SDS, leaving an intact acellular myoscaffold (44) (Fig. 3). These on-slide acellular myoscaffolds were generated from severely fibrotic dystrophin-deficient (*mdx*) muscles, healthy control muscle, and muscle overexpressing the small tetraspanin protein, sarcospan, which was previously shown to mitigate many aspects of muscular dystrophy by enhancing laminin binding (53, 54). Areas of dense scars were identified in the acellular myoscaffolds by probing for collagen III, collagen IV, collagen VI, laminin, and fibronectin. The acellular scaffolds were seeded with skeletal muscle progenitor cells. On severely fibrotic decellularized matrices, these cells exhibited decreased cell motility and migration compared with cells seeded on regions of *mdx* muscle with less fibrosis or wild-type myoscaffolds (44). The interaction between these stem cells and the matrix also differed; wild-type myoscaffolds supported better cellular adherence and the cells could be visualized as mechanically deforming the matrix. In contrast, cells cultured on severely fibrotic *mdx* acellular scaffolds were unable to deform and remodel the stiffer matrix and took on an apoptotic morphology (44). The focal regions of increased fibrosis were associated with reduced laminin remodeling and increased collagen crosslinking, and overexpression of the sarcospan laminin binding complex induced a compensatory matrisome (44). This study demonstrates the importance of spatially distinct regions in dystrophic muscle showing the role of collagen crosslinking in regulating stem cell differentiation. Concomitant with the cellular behavioral differences observed in cells seeded on fibrotic myoscaffolds, genes associated with cell adhesion, migration, and skeletal muscle maturation genes were downregulated in skeletal muscle progenitor cells seeded on *mdx* acellular myoscaffolds compared with WT scaffolds, providing cellular pathways responsive to the pathologically remodeled matrix (44).

Although a useful method, decellularization approaches may have limitations. The detergents or agents used to remove cellular material may produce nondenatured conditions for proteins causing adoption of nonnative states. However, detergent methods during electrophoresis also denature proteins yet remain useful to ascertain some protein-protein interactions. In modeling cell-matrix interactions, the time that matrices remain a suitable substrate for cell culture is a relatively short duration, and approaches to preserve stable matrix structure are needed for longer-term studies.

### Conclusions and Future Directions

Skeletal muscle fibrosis in muscular dystrophy has similarities to fibrosis after severe injury or even in aging but differs in intensity and composition. The progressive nature of fibrosis in muscular dystrophy further impairs regeneration, especially in the late stages of disease. The loss of dystrophin leads to a fragile sarcolemma, and the continual breakdown of myofibers resembles some aspects of chronic injury. The excess fibrotic deposition in muscular dystrophy is best characterized for dystrophin-mediated muscular dystrophy. The degree to which the matrices differ across unique forms of muscular dystrophy is not fully defined. The process of continual pathological matrix deposition and remodeling is an active contributor to disease progression and represents an opportunity for therapeutic intervention. Recent studies spotlight the ECM’s role and its participation in influencing cellular behavior, including myoblasts but the ECM likely influences other cell types such as fibroblasts and inflammatory cells. The diseased ECM triggers a feed-forward mechanism with healthy cells transitioning toward a profibrotic disease state with subsequent fibrosis progression. The matrix, its stiffness, and the matrisome components represent an important therapeutic target.

Future efforts will build from deep knowledge of matrisome components and their role in scaffolding regulators of matrix and cell activity. The ability to define matrix proteins during progressive fibrosis, through new advances in decellularization and other matrix-specific protocols, will help uncover proteins and pathways that drive myoblast differentiation and regeneration. Decellularization methods, especially those applied in the on-slide format and combined with genetic models, can be used to decipher specific matrix contributors to cellular repair.

## GRANTS

This study is supported by the NIH Grants AR052646, HL061322, HL167813, NS047726, NS127383, and T32HL134633.

## DISCLOSURES

Northwestern University filed provisional patent nos. 62/783,619 and 63/309,925 on behalf of the authors (A.R.D. and E.M.M.). E.M.M. has served as a consultant for Amgen, AstraZeneca, Cytokinetics, Pfizer, PepGen, and Tenaya Therapeutics and is the founder of Ikaika Therapeutics. A.R.D. is CSO at Ikaika Therapeutics. None of the other authors has any conflicts of interest, financial or otherwise, to disclose.

## AUTHOR CONTRIBUTIONS

E.M.M., A.M.L, G.L., and A.R.D. prepared figures; A.M.L., G.L., and A.R.D. drafted manuscript; E.M.M. edited and revised manuscript; A.M.L, G.L., A.R.D., and E.M.M. approved final version of manuscript.
